# Supplementary material for: Mitochondrial Analysis of Sparidae Species to Detect a New DNA Barcoding Marker for Dentex gibbosus to Utilize against Fraud
Source: Foods. 2023 Sep 15;12(18):3441. doi: 10.3390/foods12183441 (PMC10530232; doi:10.3390/foods12183441)
Supplement: Supplementary file 1 [file foods-12-03441-s001.zip › Supplementary material_290bp sequence.pdf]

Supplementary material

Results for 290bp NAD2 amplified fragment sequencing. All *D. gibbosus* NAD2 amplified fragments reported the same sequence, except for Dg1 and Dg2 from the Aegean Sea, that reported two different nucleotides (highlithed).

Dg1 and Dg2 specimens 290bp sequence:
TACATCAACCCTCATCGGAGGTTGAGGCGGCTTAAACCAAACTCAGCTGCGAAAAATTCTTGCCTACTCCT
CCATTGCTCACCTGGGCTGAATAATTCTTGTTCTGCAATTTTCCCCCTCTCTCGCCATTCTAGCCCTTACTATC
TATTTCATCATGACATTTTCAGCATTCCTCACATTCAAGCTAGTCAACTCAACCAATATTAACTCACTCGCTAT
GTCCTGATCAAAAGCTCCCATAATCACTGCCCTCGCCCCCCTCATTCTCCTATCCCTTGGAGGCTTGCCCC

Dg3-Dg10 specimens 290bp sequence:
TACATCAACCCTCATCGGAGGTTGAGGCGGCTTAAACCAAACTCAGCTGCGAAAAATTCTTGCCTACTCCT
CCATTGCTCACCTGGGCTGAATAATTCTTGTTCTGCAATTTTCCCCCTCTCTCGCCATTCTAGCCCTTACTATC
TATTTCATCATGACATTTTCAGCATTCCTCACATTCAAGCTAAACAACTCAACCAATATTAACTCACTCGCTA
TGTCCTGATCAAAAGCTCCCATAATCACTGCCCTCGCCCCCCTCATTCTCCTATCCCTTGGAGGCTTGCCCC
