# Supplementary material for: Mitochondrial Analysis of Sparidae Species to Detect a New DNA Barcoding Marker for Dentex gibbosus to Utilize against Fraud
Source: Foods. 2023 Sep 15;12(18):3441. doi: 10.3390/foods12183441 (PMC10530232; doi:10.3390/foods12183441)
Supplement: Supplementary file 1 [file foods-12-03441-s001.zip › Supplementary material_Melting temperatures.pdf]

Supplementary material

Melting Temperature obtained for each species.

**Species**	**Tm**
A. latus	79.8
A. schlegelii	79.6
D. angolensis	81.1
D. dentex	80.9
D. tumifrons	80.5
D. cervinus	79.7
D. hypselosomus	79.8
D. puntazzo	79.7
D. sargus	80
P. acarne	80.9
P. bogaraveo	80.2
P. erythrinus	80.7
P. auriga	80.2
P. caeruleostictus	81.2
P. major	79.5
P. edita	80
R. sarba	79.9
S. aurata	81
